# The Foreign Journals: Midwifery

**Published:** 1839-10

**Authors:** 


### MIDWIFERY.

### *Singular Case of a Woman delivered of Five Children.*

Giuseppa Califani, of Naples, at the age of fourteen years and three months, was married to a man aged twenty-seven, by whom she had ten children at eight accouchements; at the fifth and sixth producing twins. She lived with her husband ten years, and remained a widow three years after his death; she then took a second husband, whose age was about twenty-nine. After two regular accouchements, upon her third pregnancy she became enormously large; so that, at seven months, she appeared to be at the termination of her natural period. She was taken, however, at seven months, with labour-pains, and brought forth successively, and by natural presentations, five living children, all of whom were baptized. The mother did not suffer anything extraordinary. Four of these children were females, and one male. The male infant was delivered first, and, after a few minutes, one female; then, after a cessation of fifteen minutes' interval between each, the other three followed. The infants much resembled each other, and were of a regular form, and well grown, and very nearly of the ordinary size of a seven months' fœtus; each weighed about 3½lbs., and measured in length a French foot. The insertion of the umbilical cord was about four lines lower down than ordinarily. The placentas with their membranes were four instead of five; and each had its proper umbilical cord, except the fourth, which contained two in one large sac. The fœtus, with their membranes, placenta, and umbilical cords, are preserved in the Royal Anatomical Museum of the University of Naples. Vincenzo Licci, of Calimera, in Otranto; Vincenzo Massari, of Molfetta, in Bari; and Dr. Antonio Scacani of Naples, conducted the examination.

[The above case was originally reported in the *Giornale delle due Sicilie*, June 28, 1838, by Dr. Pasquale Cattolica, Clinical Professor of Midwifery, and Sig. Antonio Nanula, Professor of Pathology in the University of Naples—and bears every mark of authenticity: we extract it from the under-mentioned journal.] *Bulletino delle Scienze Mediche. Agosto e Settembre,* 1838.

---

### *On Morbid Accumulation of the Liquor Amnii in Pregnant Women.*
By Dr. Bunsen, of Frankfort on the Maine, and Dr. Kyll, of Cologne.

Case i. Mrs. C———, a healthy woman, aged twenty-six years: when she first came under Dr. B.'s notice, in the spring of 1826, she was pregnant for the ninth time. All her labours had taken place at the end of the full term of pregnancy, and her first five infants were born alive and healthy; her sixth and seventh children, however, were born dead and putrid; and the expulsion of the eighth, which was very imperfectly developed, was accompanied by the escape of a very large quantity of liquor amnii. The patient arrived safely at the end of her ninth pregnancy, but the size of her abdomen was unusually great, and the motions of the fœtus were remarkably feeble. Labour came on, on the 6th of September, 1836; and no sooner did the membranes rupture, than an enormous quantity of liquor amnii escaped (twelve pints), together with the fœtus, the development of which appeared to have been arrested about the sixth month. The placenta weighed two pounds and a half; its structure was very spongy, which peculiarity, Bunsen remarks, he has always found coexisting with arrested development of the fœtus, and unusually abundant secretion of the liquor amnii. In March, 1827, Mrs. C. again became pregnant, and her abdomen once more attained a very large size, but the motions of the child were very distinct. On December 25, 1837, labour pains began; the membranes, when they ruptured, gave exit to nineteen pints of liquor amnii; the child was hydrocephalic, and its abdomen was so distended by fluid that, but for the large size of the mother's pelvis, its extraction alive would have been impossible. The placenta weighed a pound and three quarters. The child survived only forty-eight hours, but the patient recovered, as after her former labours, with great rapidity.

Having become pregnant again in the winter of 1829, the patient placed herself under Dr. Bunsen's care as soon as she felt the motions of the child; and from that time till her delivery, which took place at the end of the eighth month owing to over exertion, she made use of mild diuretics, wore a bandage round the abdomen, and took regular exercise. Labour commenced in the evening of November 24th; on the following morning the membranes ruptured, but the quantity of liquor amnii which flowed away was not unusually great. The child was malformed and labouring under a great degree of ascites, it gave but feeble signs of life, and soon expired. The placenta was again unusually large. During her next pregnancy, which took place in 1831, Mrs. C. did not consult Dr. Bunsen, who was not aware of her being pregnant, till he was summoned to attend her in labour on the 29th of September. The uterine action was energetic, and the patient soon gave birth to a feeble child, not more developed than children usually are at the sixth month, the expulsion of which was accompanied with the escape of a larger quantity of liquor amnii than in any preceding labour. The placenta weighed three pounds and a half. The feeble infant lived but for a month, and Mrs. C. did not again become pregnant.

CASE II. The next case related is that of a healthy woman who had thrice given birth to dead children, each labour having been accompanied by the escape of a very large quantity of liquor amnii. At the end of her fourth pregnancy she consulted Dr. Bunsen, on account of anasarca, with œdema and erysipelatous affection of the labia; and while under his care she gave birth, after a difficult labour, on April 16, 1831, to a female child, affected with ascites, and which lived only twenty hours. The quantity of liquor amnii was very great, and the placenta unusually large. The patient's health continued indifferent for some time after her delivery, but she gradually recovered under the use of steel; and becoming pregnant for the fifth time at the end of 1831, she again applied to Dr. Bunsen. Gentle diuretics with ether and bitters were prescribed; and on October 3, 1832, a healthy male child was born; nor was there anything unnatural in the state of the placenta, or in the quantity of the liquor amnii. Dr. B. has been unable to learn the subsequent history of this patient.

CASE III. A case follows, in which dropsical affection of the mother was unattended by any similar condition of the fœtus. The patient gave birth to three healthy children in the space of four years, though suffering all the time from dropsical symptoms, which at last proved fatal, a few days after her third confinement.

[We cannot but regret that Dr. Bunsen has given no account of the dissection of the children in these cases, since, had he done so, the value of these observations would have been greatly enhanced.]

CASE IV. The patient, a lady, twenty-eight years old, first came under Dr. Kyll's care in consequence of having been infected with syphilis, by a girl whom she had employed to draw her breasts, after her first confinement. After having suffered from this disease for eight months, she applied to Dr. Kyll, who prescribed the corrosive sublimate with advantage; but, when nearly well, she aborted at the third month of her second pregnancy. Three months afterwards, having perfectly recovered, she became again pregnant, and suffered much during this pregnancy from varicose veins of the thighs; venesection, however, afforded her great relief. At the end of the sixth month, without any assignable cause, the liquor amnii began to drain away; two days after which labour set in, and a female child was born, which struggled a little and then died. The expulsion of the child was accompanied with the escape of a very large quantity of liquor amnii. At the expiration of two hours, the placenta, which was universally adherent, was removed, when Dr. Kyll was struck by its remarkably large size. The circumference of the organ was more than a third greater than natural, and its thickness was double that of an ordinary placenta. It was of a pale red colour and spongy structure; but on dividing it, its tissue appeared perfectly natural, save that the blood-vessels were larger than usual, as were also the umbilical arteries and veins, although the child wanted three months of the full term. Three days after delivery, the patient lost a considerable quantity of blood from the uterus, but eventually she perfectly recovered. The large size of the abdomen of the fœtus had already attracted Dr. Kyll's attention; and on making an examination of it, a large quantity of straw coloured fluid was found in its cavity and between the folds of the omentum. The liver was very large, occupying the whole abdomen, and reaching downwards nearly to the bladder; but its substance, when cut into, presented no sign of inflammation, nor any other change in structure than great development of its vessels. This unusually large size is referred, by Dr. K , to the hypertrophy of the placenta and the consequently increased quantity of blood which the liver would receive. The enlargement of the placenta is, in his opinion, owing rather to congestion than to inflammation, since the results of inflammation are obliteration of vessels from exudation, and consequently diminished nutrition of the organ, owing to which it shrinks, and its structure becomes more compact and firmer than natural, sometimes attaining an almost cartilaginous hardness. On making a section of a portion of inflamed placenta, the fœtal surface is found of a straw colour, which gradually changes to a grayish red, and then to a dark red colour on approaching the uterine surface. Inflammation involves some portions only of the placenta, while hypertrophy extends to the whole organ, which is increased in all its dimensions; its vessels are often enlarged, and its tissue is rendered spongy and easily lacerable, though neither infiltration nor hepatization of its substance exists. *Neue Zeitschrift für Geburtskunde.* Band vii. Heft i.

---

*Galvanic Obstetric Forceps.* By Prof. Kilian, of Bonn.

Desirous of ascertaining how forceps, the blades of which were made of different metals, would affect the uterus in tardy labours, Professor Kilian caused such a pair to be manufactured. An opportunity soon occurred for testing its efficacy. Labour was proceeding slowly, from deficient action of the uterus, and for two hours and a half the head had remained in the same spot. The forceps were applied, and, as soon as the two blades were joined, the uterus was felt to contract powerfully, but not in such a manner as to assist in the expulsion of the child. [Was not this mere accident?] *Medicinische Zeitung.* No. xii. 1839.

---

*Singular case of Malformation.*

On the 20th March of this year (1839), at Schneidemühl, a stout woman, who had before borne five living and healthy children, was delivered of a remarkably stout female infant, presenting the following defective formation. The heart, and, under it, the stomach, both organs apparently separated by a partition, lie outside the thoracic and abdominal cavities, in a sack of skin nearly transparent. The protrusion is through a deficiency of the lower third of the sternum and upper part of the wall of the abdomen, as far as midway between the pit of the stomach and navel. The fissure is altogether 5½ inches long, and 2¼ inches broad, and is situate almost in the middle line of the body. The child is living, sucks, and is otherwise well. The prolapsed parts are protected by an appropriate bandage.

*Med. Zeitung. April* 17, 1839.

### *Case of Mole Pregnancy with Spontaneous Amputation.* By Dr. SCHWABE, Stadtphysicus, &c. at Cölleda.

THIS was the patient's fifth pregnancy. After much incautious exertion, &c, pains came on with considerable hemorrhage, so that the ovum was supposed to have come away among the coagula. A frequent draining of bloody serum followed, and the hemorrhage returned very profusely so as to reduce her seriously. The uterus was found as big as a large fist, above the symphysis pubis, the breasts flaccid, the vagina relaxed, the os uteri very high up, somewhat backwards, soft and closed: bark was given, and afterwards acetate of lead and opium, with very good effect. The hemorrhage, however, returned so profusely as to threaten dissolution: all attempts to dilate the os uteri mechanically being fruitless, repeated doses of ergot were given; and on the fourth day, slight pains in the abdomen made their appearance, and soon became regular, and expelled a hollow mole without any considerable loss. It contained a fœtus of about three months, the surface of which, as well as the inside of the amnion, was covered with a firm reddish substance. The cord formed a knot round the right leg at the ankle, and had nearly separated the foot from it: immediately above the ligature was an enlargement somewhat greater than the foot itself, and resembling a clump-foot in form, and adhering to the left knee.

[We quote this case for the purpose of recording additional testimony in confirmation of Dr. Montgomery's valuable observations upon this subject, to which we were obliged to allude but briefly in our review of his work on the Signs of Pregnancy.] *Siebold's Journal.* B. xvii. St. 2. 1838.

---

### *Case of Spontaneous Evolution.* By Dr. CARGANICO.

[THE following case is interesting, as it tends to confirm the original views of Dr. J. Douglas, of Dublin, upon this subject, to whom we are indebted for having first pointed out the real mechanism of this peculiar mode of expulsion.]

The patient was a robust, healthy primipara, æt. twenty-eight. The membranes had ruptured with the first pains, and an arm prolapsed. No assistance was requested for two days, when two midwives tried to turn, but did not succeed. The pains were violent, and the author was summoned. He found the patient suffering under incessant and painful uterine contractions, without a moment's cessation. The skin was hot, the pulse small and hard, the patient pale and exhausted. The arm (the left) was immensely swollen, livid and black, and the epidermis peeled off. The labia and vagina were much swollen and were beginning to be dry. The author made an attempt to turn, but could not succeed; he therefore bled her, and used narcotics both inwardly and outwardly, by which means the pains in the loins and abdomen abated; she became quiet and fell into a sound sleep which lasted from five to six hours, during which she perspired freely. Slight contractions of the uterus, and almost without pain, now came on; and by their action, the arm and shoulder were detruded still further, while at the same time the left side of the thorax began to press downwards from the sacrum towards the left arm: the hip followed the side of the trunk, and, as the child turned completely round, the nates followed, and together with the trunk and legs were now expelled. The head with the arm stretched along it followed without any difficulty, as also did the placenta. The whole process lasted only a few minutes, and the patient assured the author that she had scarcely felt anything of the pain. The child, which was dead, was full grown: it was emphysematous, the epidermis peeling off, and the limbs quite flaccid. It was to this state of perfect flaccidity, the result of incipient decomposition, that the patient was indebted for the perfect ease with which the whole was accomplished.

*Medizinische Zeitung.* Nos. 29-34. *Jahrgang* 7, 1838.

*Case of Difficult Delivery of Twins which were united by the Breast.* By the Berg-chirurgus Rath of Zellerfeld. (*From the Archiv. der Harzer Chirurgen-Verein.*)

The patient was turned forty years of age; mother of several children. She had passed the first half of her pregnancy in perfect health: but during the last few weeks before labour, complained of constant cramps and much pain from the movements of the child. Shortly after the os uteri was dilated, the pains subsided; and although they were again excited by the ergot, they had no effect in forwarding the labour. The forceps was now applied, and the head brought down as far as the os externum, but beyond this it was impossible to move it; and on further examination, a second head was found behind the first in the pelvic cavity. The patient was much exhausted, the abdomen tympanitic, very pendulous, and acutely painful; the external parts much swollen. A prolapsed umbilical cord was felt beating at the lower and posterior part of the vagina; but the patient had not felt any movement for some time. As there was no doubt that the first child was dead, and that it was an insurmountable obstacle to the delivery of the other, which might still be alive, and also increased the patient's danger, by protracting the labour, the head was separated from the body with some difficulty, owing to the closeness with which it was embraced by the external parts. It was effected by means of a gum-lancet, which the author considers to be an excellent instrument for the purpose. The second head could now be reached with the forceps, and was brought down as far as the chin and nape of the neck; but another obstacle now showed itself: after repeated examinations it was ascertained that the children were united by the breast, and that one had its back to the pubes, the other to the sacrum. The author endeavoured to give them such a direction that they should be in the longest diameter of the pelvis, by which means he succeeded in bringing them down as far as the shoulders. Having carefully disengaged the arms, and turned the children again into the antero-posterior diameter, and having fixed a blunt hook upon the band which united them, he gave it to an assistant, and then, with a fore-finger in the axilla of each child, he completed the delivery. Considerable hemorrhage required the speedy removal of the placenta, and the uterus now contracted. The children (two girls) weighed 15 lbs.; they were 17 inches long. The part by which they were united was 9 inches long and 3 broad, and extended from the upper extremity of the sternum to the navel, into which one umbilical cord, which was common to both, entered. The diameter of the two children, when laid close together, was between 7 and 8 inches, from back to the other. One child had two thumbs on the right hand. The cord was 19 inches long, and unusually thick. After suffering some time from peritonitis, &c. the patient recovered.

*Siebold's Journal.* B. 17. Heft ii. 1838.

